# Effect of exercise therapy on lipid profile and oxidative stress indicators in patients with type 2 diabetes

**DOI:** 10.1186/1472-6882-8-21

**Published:** 2008-05-13

**Authors:** Lorenzo A Gordon, Errol Y Morrison, Donovan A McGrowder, Ronald Young, Yeiny Terry Pena Fraser, Eslaen Martorell Zamora, Ruby L Alexander-Lindo, Rachael R Irving

**Affiliations:** 1 Department of Medicine, University of the West Indies, Kingston 7, Jamaica; 2 Department of Basic Medical Sciences, University of the West Indies, Kingston 7, Jamaica; 3 Department of Pathology, University of the West Indies, Kingston 7, Jamaica; 4 Faculty of Pure and Applied Science, University of the West Indies, Kingston 7, Jamaica; 5 Department of Saturnino Lora Hospital, Santiago de Cuba, Cuba

## Abstract

**Background:**

Yoga has been shown to be a simple and economical therapeutic modality that may be considered as a beneficial adjuvant for type 2 diabetes mellitus. This study investigated the impact of Hatha yoga and conventional physical training (PT) exercise regimens on biochemical, oxidative stress indicators and oxidant status in patients with type 2 diabetes.

**Methods:**

This prospective randomized study consisted of 77 type 2 diabetic patients in the Hatha yoga exercise group that were matched with a similar number of type 2 diabetic patients in the conventional PT exercise and control groups. Biochemical parameters such as fasting blood glucose (FBG), serum total cholesterol (TC), triglycerides, low-density lipoprotein (LDL), very low-density lipoproteins (VLDL) and high-density lipoprotein (HDL) were determined at baseline and at two consecutive three monthly intervals. The oxidative stress indicators (malondialdehyde – MDA, protein oxidation – POX, phospholipase A2 – PLA2 activity) and oxidative status [superoxide dismutase (SOD) and catalase activities] were measured.

**Results:**

The concentrations of FBG in the Hatha yoga and conventional PT exercise groups after six months decreased by 29.48% and 27.43% respectively (P < 0.0001) and there was a significant reduction in serum TC in both groups (P < 0.0001). The concentrations of VLDL in the managed groups after six months differed significantly from baseline values (P = 0.036). Lipid peroxidation as indicated by MDA significantly decreased by 19.9% and 18.1% in the Hatha yoga and conventional PT exercise groups respectively (P < 0.0001); whilst the activity of SOD significantly increased by 24.08% and 20.18% respectively (P = 0.031). There was no significant difference in the baseline and 6 months activities of PLA2 and catalase after six months although the latter increased by 13.68% and 13.19% in the Hatha yoga and conventional PT exercise groups respectively (P = 0.144).

**Conclusion:**

The study demonstrate the efficacy of Hatha yoga exercise on fasting blood glucose, lipid profile, oxidative stress markers and antioxidant status in patients with type 2 diabetes and suggest that Hatha yoga exercise and conventional PT exercise may have therapeutic preventative and protective effects on diabetes mellitus by decreasing oxidative stress and improving antioxidant status.

**Trial Registration:**

Australian New Zealand Clinical Trials Registry (ANZCTR): ACTRN12608000217303

## Background

Diabetes mellitus is a worldwide health problem predisposing to markedly increased cardiovascular mortality and morbidity [1]. Lipid abnormalities significantly contribute to the increased risk of cardiovascular disease and other morbidity in diabetics [2]. There is a growing body of evidence showing that hyperglycaemia and dyslipidaemia are linked to increased cardiovascular risk [3]. It has been demonstrated that high levels of serum TC, triglycerides, LDL, VLDL, glycated haemoglobin (HbA_1c_), microalbuminuria, hypertension, low concentration of HDL and increased body mass index (BMI) are significantly associated with coronary heart disease [4].

Oxidative stress induced by reactive oxygen species (ROS), which is generated by hyperglycaemia, is one of the major foci of recent research related to diabetes mellitus [5]. Diabetes mellitus is characterized by hyperglycaemia together with biochemical alterations of glucose and lipid peroxidation [6]. There are several studies that have evaluated free radical induced lipid peroxidation and the antioxidants in diabetic patients [7,8]. Some complications of diabetes mellitus are associated with increased activity of free radical-induced lipid peroxidation and accumulation of lipid peroxidation products [9]. Mechanisms that contribute to increased lipid peroxide formation in diabetic patients include: hyperglycaemic-induced glucose auto-oxidation, non-enzymatic glycation of proteins and lipids, increased sorbitol pathway activity, oxidation of advanced glycation end-products (AGEs) and cyclooxygenase dependent formation of prostaglandin H2 (PGH_2_) [10]. A variety of natural antioxidants exist to scavenge oxygen free radicals and prevent oxidative damage to biological membranes. One group of these antioxidants is enzymatic (intracellular), which includes superoxide dismutase (SOD), glutathione peroxidase and catalase [11]. In addition to enzymatic antioxidants, are the major natural antioxidants, most of them derived from natural sources by dietary intake and include vitamin A, vitamin C, vitamin E and carotenoids [12]. Abnormally high levels of peroxidation and the simultaneous decline of antioxidant defense mechanisms can lead to damage of cellular organelles and oxidative stress [13].

Exercise is a major therapeutic modality in the treatment of diabetes mellitus [14]. Regular physical exercise has been reported to be effective in the prevention and delay of onset of type 2 diabetes, increases insulin sensitivity, and ameliorates glucose metabolism [15]. Yoga has become increasingly popular in Western cultures as a means of exercise and training fitness [16]. It has been used clinically as a therapeutic intervention and its practice includes muscle stretching, breathing exercises, behavioural modification, and dietary control through mental discipline [17]. A growing number of research studies have shown that Hatha yoga can improve strength and flexibility, and may help control physiological variables such as blood pressure, respiration and heart rate, and metabolic rate to improve overall exercise capacity [18,19]. Studies carried out on medium or long-term effect of yoga exercise on oxidative stress parameters and antioxidant status in type 2 diabetic patients are sparse. The aims of the present study were therefore to investigate the effects of Hatha yoga as well as conventional physical training (PT) exercise interventions on lipid profile, oxidative stress parameters and antioxidant status after 3 and 6 months of intervention in patients with type 2 diabetes mellitus.

## Methods

This prospective randomized control study was conducted at The National Institute of Endocrinology and The "Hermanos Ameijeiras" Hospital, Havana, Cuba from September 1998 to February 1999. The patients were selected according to the CONSORT declaration [20] and include only those with type 2 diabetes who had been trained in diabetes education and instruction, exercise, diet and medication according to the recommendations of the International Diabetes Federation (IDF), for a minimum of 3 months, and who met the following criteria for the study. These criteria were: type 2 diabetes mellitus without malnutrition or severe complications of the disease (cardiovascular, renal, visual and cerebral), between 40–70 years old, duration of the disease between 1–10 years, good psychological condition (in accordance with the psychologist's consideration), non-smoker and non-alcoholic. The study was approved by The "Hermanos Ameijeiras" Hospital ethics committee and informed consent was obtained from all patients who participated in the study.

The managed groups (Hatha yoga and conventional PT exercise) were trained for 24 weeks in basic exercise techniques, diabetes education and instructions. The yoga class was designated by a certified Hatha yoga instructor and a physician. The programmes were carefully illustrated through workshops and subjects were required to attend one yoga class weekly for twenty four weeks along with home exercise. None of the subjects in the Hatha yoga group were exposed to yogic practices. Each yoga session consisted of 20 minutes of pranayamas (breath-control exercises), 25 minutes of dynamic warm-up exercises, 60 minutes of asanas (yogic postures), and 15 minutes of supine relaxation in savasana (corpse pose). Subjects were given a booklet illustrating the specific pose to help with their independent practice [21].

Subjects in the conventional exercise PT exercise group also attended classes and were engaged mainly in aerobic exercise for 2 hours. A certified exercise instructor directed the conventional PT exercise intervention arm of the study. The conventional PT exercises consisted of one class per week for 24 weeks along with home exercise. The conventional PT sessions consisted of 15 minutes of warm up exercises, 30 minutes of aerobic walking on an outdoor 400-meter track, 20 minutes of body flexibility exercises, 20 minutes of aerobic dance, 25 minutes of games and 10 minutes of warm down exercises. Daily home exercise 3 – 4 times per week for 1 hour in the same Rate of Perceived Exertion was encouraged for subjects in both the Hatha yoga and conventional PT exercise classes. Intensity of exercise was determined by measurement of the pulse rate, and heart rate before, during and after exercises in subjects in the Hatha yoga and conventional PT exercise groups. Target heart rate was initially estimated as 70% of maximum based on morning resting heart rate and age. The measurement of perceived exertion during and immediately following exercise was done in the Hatha yoga and conventional PT groups by using the modified Borg Rate of Perceived Exertion Scale [22]. A questionnaire with statements as given in Borg's rating scale was used to assess the magnitude of exertion during and immediately after exercise. The Perceived Exertion Scale consisted of statements between 6 and 20 ('no exertion at all' to 'maximum exertion'). Subjects were instructed to exercise at a level of 8 – 10 on the Perceived Exertion Scale. Each managed group fulfilled a specific weekly programme that included the following: medical and psychological evaluation of patients and instructions on: education, diet, specific treatments, personal care and exercises (conventional PT or Hatha yoga). Subjects were also given a booklet in which they noted dietary compositions, medications taken daily, signs and symptoms, daily blood pressure, weekly glycaemia and information on adherence to home-based exercise programmes. Compliance with the interventions was assessed by having study participants complete daily 1-week log sheets that recorded whether or not they exercised or practiced yoga and for how long. The booklets were reviewed weekly by personnel blinded to the study. Class attendance was also recorded. The control group followed a treatment plan as recommended by their clinics or general physicians and was never seen by the personnel of this study for diabetes management. They were not engaged in any kind of active exercise intervention during the entire study period. All subjects were encouraged to see their attending physician regularly.

**Figure 1:**
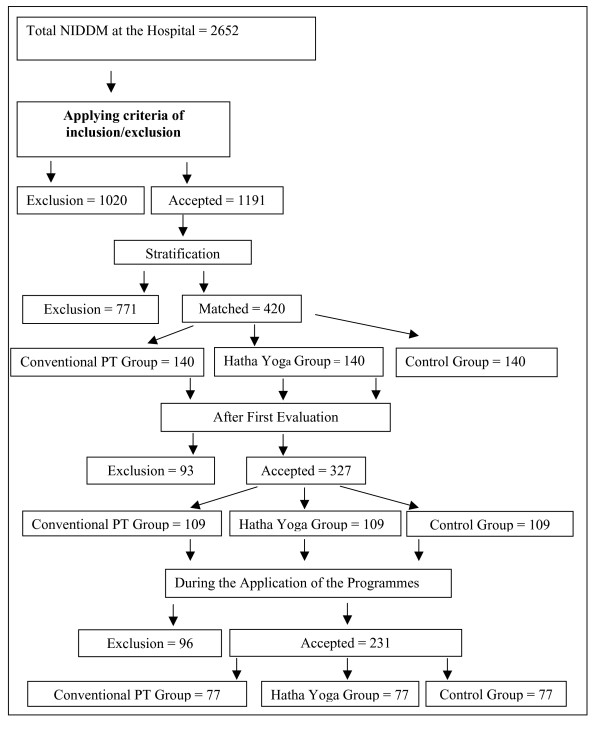
Schematic diagram representing the study design.

Figure 1 gives a description of the selection of type 2 diabetic patients in the study. A total of 231 subjects were recruited for this prospective randomized study: 77 type 2 diabetic patients in the Hatha yoga exercise group (62 females and 15 males) that were matched with 77 type 2 diabetic patients in the conventional PT exercise group (62 females and 15 males) and another 77 type 2 diabetic patients serving as the control group (62 females and 15 males). All 231 patients completed the study.

Measurement of the weekly variations of clinical and metabolic parameters was used to check the effectiveness of both interventions (Hatha yoga exercise or conventional PT). Baseline assessment of outcome measures and parameters were performed before the subjects were randomized and occurred 1 to 14 days before classes started. On the baseline visit, medical history was reviewed, demographic data were recorded, and blood samples taken for biochemical investigations. Blood was drawn from an antecubital vein at baseline, 3 months and 6 months for biochemical investigations, in post absorptive state. The blood was drawn between 7:30 am and 9:00 am, without stasis, and the serum was separated within an hour of collection. On the day of the blood collection, subjects were asked to abstain from Hatha yoga or conventional PT exercises. The biochemical investigations that were determined included lipid profile – serum TC, triglyceride, HDL, LDL and VLDL. Fasting blood glucose was measured before exercises. Oxidative stress indicators included: concentration of MDA, PLA2 activity and POX. The antioxidant status parameters were SOD and catalase activities.

All laboratory determinations were carried out at the "Hermanos Ameijeiras" Hospital. The personnel of the laboratories were blinded to the study. The fasting blood glucose concentration for each group was determined by using the Reflolux S type 1172115 glucometers (Boehringer Mannheim, Germany) [23]. This is based on the glucose-oxidase method [24]. Fasting venous blood was taken from each subject at baseline, at the third and sixth months. Total cholesterol, triglyceride and HDL were determined by enzymatic methods [25]. High density lipoprotein was measured after precipitating VLDL and LDL cholesterol in the presence of magnesium ions. The LDL fraction was calculated by the Friedwald formula [26].

Malondialdehyde concentration in the serum was measured spectrophotometrically according to Yagi [27] and PLA2 activity in serum was determined according to the method described by Lobo and Radbani [28]. Protein oxidation is based on the detection of the carbonyl group that appears as a result of the oxidation of lateral chains of certain amino acids. Plasma carbonyl group levels were evaluated following the 2,4-dinitrophenylhydrazine (2,4-DNP) assay [29], and were expressed as nanomoles per milligram of protein. The protein concentration was determined using bovine serum albumin as standard, according to Bradford's method [30]. The SOD activity was determined by inhibition of pyrogallin (formed due to auto-oxidation of pyrogallol) [31] and catalase activity was measured by ultraviolet method, based on the transformation of hydrogen peroxide [32].

The data were collected and recorded on questionnaires, and a database was developed in Microsoft Excel 2000. The calculations were carried out with the soft-wares EPI-INFO Version 6.0 and SPSS Version 10.0 with a level of statistical significance of 95%. The individual variables were evaluated to determine the changes in the three groups (Hatha yoga, conventional PT and control) during the different periods (baseline, third or sixth month). To investigate the effects of a variable within a group the Within – Subjects factors analysis, and the effects between the groups Between – Subjects analysis were used according to Two Way ANOVA with a 95% confidence interval. Two Way ANOVA was applied to detect differences among groups (Hatha yoga, conventional PT exercise and control) and within groups over the duration of treatment. According to the behaviour of the variables, each table was represented by the mean and the standard error of the mean (SEM). As the three groups were of equal sizes then the Tukey's Honestly Significant Difference (Tukey HSD, available in SPSS) was used for posthoc comparisons. Differences were considered significant if P < 0.05.

## Results

There were 231 type 2 diabetic patients, 186 (80.5%) females and 45 (19.5%) males. Subjects matched according to age and sex and the mean ages were very similar in the three groups according to gender (Table 1). There was no significant gender-specific difference in demographic and biochemical parameters measured in the study. All 231 patients completed the study by participating in the 6-month assessment. Compliance with home-based exercise in the Hatha yoga and conventional PT groups was in the range from 80 – 85% and averaged attendance at classes was 90 – 95%. In the Hatha yoga and conventional PT exercise groups there were significantly decreased concentrations of FBG at the third (P < 0.0001) and sixth months of exercise (P < 0.001) compared to baseline values (Table 2). The concentrations of FBG in the Hatha yoga and conventional PT exercise groups decreased by 29.48% and 27.43% respectively compared to a reduction of 7.48% in the control group. There were significant differences in FBG between the managed exercise groups and the control group at the third and sixth months (P < 0.0001).

**Table 1:** Frequency of treated type 2 diabetic subjects according to group, sex and age

Group	Female			Male			Total		
	No.	%	Mean Age	No.	%	Mean Age	No.	%	Mean Age
Conventional	62	80.5	63.5	15	19.5	65.6	77	100.0	63.9
Hatha yoga	62	80.5	63.8	15	19.5	64.7	77	100.0	64.0
Control	62	80.5	63.8	15	19.5	62.5	77	100.0	63.6

**Table 2:** Comparison of FBG, cholesterol and triglyceride concentrations amongconventional PT exercise, Hatha yoga exercise and control type 2 diabeticpatients over a 6-month period

Variable/Group	Baseline	3 Months	6 Months
FBG (mmol/L)			
Conventional PT	11.74 ± 0.34	8.71 ± 0.24**^ab^	8.52 ± 0.24**^ab^
Hatha yoga	11.84 ± 0.45	8.58 ± 0.44**^ab^	8.35 ± 0.44**^ab^
Control	11.77 ± 0.44	12.79 ± 0.37	10.89 ± 0.39
Cholesterol (mmol/L)			
Conventional PT	4.32 ± 0.13	4.29 ± 0.14**^ab^	4.27 ± 0.14**^ab^
Hatha yoga	4.39 ± 0.14	4.33 ± 0.16**^ab^	4.33 ± 0.15**^ab^
Control	4.37 ± 0.13	4.93 ± 0.14	5.11 ± 0.13
Triglyceride (mmol/L)			
Conventional PT	1.83 ± 1.11	1.78 ± 1.10	1.70 ± 1.11
Hatha yoga	1.83 ± 1.13	1.76 ± 1.14	1.72 ± 1.13
Control	1.81 ± 1.13	1.83 ± 1.12	1.90 ± 1.12

Values represent Mean ± S.E. **p < 0.0001; a: significantly different from baseline; b: significantly different from control.

There was a significant reduction in serum TC in the Hatha yoga and conventional PT exercise groups at the end of six months (P < 0.0001). In the Hatha yoga exercise group the TC concentration decreased from 4.39 ± 0.14 mmol/L to 4.33 ± 0.15 mmol/L, whilst in the conventional PT exercise group it decreased from 4.32 ± 0.13 mmol/L to 4.29 ± 0.14 mmol/L. The mean concentrations of TC significantly differed between the managed exercise groups and the control group (P < 0.001) and in latter group it increased from 4.37 ± 0.13 mmol/L at baseline to 5.11 ± 0.13 mmol/L after 6 months. The concentration of serum triglyceride was not significantly decreased in the managed exercise groups, with a reduction of 6.0% and 7.1% in the Hatha yoga and conventional PT exercise groups respectively (P = 0.432). The concentration of serum triglyceride in the control group increased by 5.0% and there was no significant differences between this group and the managed exercise groups (P = 0.068; Table 2).

No significant differences were found in the concentrations of LDL and HDL in the Hatha yoga and conventional PT exercise groups after six months when compared with baseline value or control group (Table 3; P < 0.05). The concentrations of VLDL in the managed group significantly differed at the sixth month from baseline values. In the Hatha yoga and conventional PT exercise groups the concentrations of VLDL decreased from 0.83 ± 0.05 mmol/L at baseline to 0.77 ± 0.05 mmol/L after six months (P = 0.036). However, there was no significant difference in the concentration of VLDL in the managed exercise groups and the control group (P = 0.788).

**Table 3:** Comparison of HDL, LDL and VLDL among conventional PT exercise, Hathayoga exercise and control type 2 diabetic patients over a 6-month period

Variable/Group	Baseline	3 Months	6 Months
HDL (mmol/L)			
Conventional PT	0.93 ± 0.04	0.94 ± 0.04	0.94 ± 0.04
Hatha yoga	0.94 ± 0.04	0.97 ± 0.04	0.97 ± 0.05
Control	0.93 ± 0.93	0.93 ± 0.04	0.91 ± 0.04
LDL (mmol/L)			
Conventional PT	3.02 ± 0.12	3.00 ± 0.12	3.00 ± 0.12
Hatha yoga	3.09 ± 0.14	3.01 ± 0.15	3.01 ± 0.14
Control	3.07 ± 0.12	3.17 ± 0.13	3.24 ± 0.12
VLDL (mmol/L)			
Conventional PT	0.83 ± 0.05	0.78 ± 0.05	0.77 ± 0.05*^a^
Hatha yoga	0.83 ± 0.07	0.79 ± 0.07	0.77 ± 0.07*^a^
Control	0.84 ± 0.06	0.84 ± 0.06	0.84 ± 0.06

Values represent Mean ± S.E. *p < 0.05; a: statistically significant different from baseline.

Lipid peroxidation as indicated by MDA significantly decreased in the managed exercise groups after six months, with reductions of 19.9% and 18.1% in the Hatha yoga and conventional PT exercise groups respectively (P < 0.0001). There were significant differences in the concentrations of MDA between the managed exercise groups and the control group (P = 0.004), with no change in this parameter for the latter group after six months (Table 4).

**Table 4:** Comparison of MDA, PLA2 and POX concentrations among conventional PTexercise, Hatha yoga exercise and control treated type 2 diabetic patients over a6-month period

Variable/Group	Baseline	3 Months	6 Months
MDA (nmol/L)			
Conventional PT	2.32 ± 0.12	2.23 ± 0.12	1.90 ± 0.10**^a*b^
Hatha yoga	2.36 ± 0.20	2.21 ± 0.15	1.89 ± 0.16**^a*b^
Control	2.35 ± 0.12	2.36 ± 0.12	2.37 ± 0.13
PLA2 (IU)			
Conventional PT	1.97 ± 0.08	2.19 ± 0.08	2.29 ± 0.09
Hatha yoga	2.10 ± 0.08	2.12 ± 0.07	2.25 ± 0.07
Control	2.06 ± 0.09	2.16 ± 0.09	2.15 ± 0.09
POX (nmol/mg)			
Conventional PT	2.25 ± 0.12	2.21 ± 0.14	2.34 ± 0.15
Hatha yoga	2.19 ± 0.13	2.20 ± 0.14	2.34 ± 0.13
Control	2.21 ± 0.13	2.23 ± 0.15	2.25 ± 0.16

Values represent Mean ± S.E. **p < 0.0001; *p < 0.05; a: significantly different from baseline; b: significantly different from control.

The activity of PLA2 in the Hatha yoga exercise group increased by 7.1% and markedly increased by 16.2% in the conventional PT exercise group after six months (P > 0.05). There was no significant difference in activity of PLA2 between the managed exercise groups and control group (P = 0.057). No significant difference was found in the concentration of POX in the managed exercise groups after six months or when compared with the control group (Table 4).

The activity of SOD significantly increased by 24.08% and 20.18% in the Hatha yoga and conventional PT exercise groups respectively (P = 0.031; Table 5), while it decreased by 5.35% in the control group after six months. There was no significant difference in the activity of SOD in the managed exercise groups compared to the control group (P = 0.118) after six months. The activity of catalase increased by 13.68% and 13.19% in the Hatha yoga and conventional exercise groups respectively (P = 0.144), but their differences with those of the control group were not statistically significant (P = 0.744).

**Table 5:** Comparison of the activities of superoxide dismutase (SOD) and catalaseamong conventional PT exercise, Hatha yoga exercise and control type 2 diabetic patients over a 6-month period

Variable/Group	Baseline	3 Months	6 Months
SOD (U/mL)			
Conventional PT	11.25 ± 0.86	11.66 ± 0.82	13.52 ± 0.96*^a^
Hatha yoga	11.17 ± 1.18	11.64 ± 1.15	13.86 ± 1.11*^a^
Control	11.01 ± 1.05	11.03 ± 0.98	10.42 ± 0.93
Catalase (U/mL)			
Conventional PT	80.28 ± 5.57	85.44 ± 5.54	90.87 ± 5.13
Hatha yoga	80.36 ± 7.02	85.53 ± 3.77	91.35 ± 5.21
Control	82.10 ± 5.89	81.44 ± 4.67	82.11 ± 5.34

Values represent Mean ± S.E. *p < 0.05; a: significantly different from baseline.

## Discussion

The present study confirmed the positive effects of Hatha yoga as well as conventional PT exercises on biochemical, oxidative stress and oxidant status in type 2 diabetics over six months. Type 2 diabetic patients engaged in Hatha yoga exercise and conventional PT exercises demonstrated lower fasting blood glucose, serum TC and VLDL concentrations. Malondialdehyde concentration, a lipid peroxidation product and marker of oxidative stress significantly decreased. The activities of SOD an enzymatic oxidant significantly increased and this was accompanied by a non-significant increase in that of catalase. The results of this study are similar to that of Bijlani et al. where yoga significantly decreased FBG, serum TC, LDL, VLDL, triglycerides and TC/HDL ratio in individuals attending a lifestyle education based program for 9 days [33]. Other researchers have reported that yoga sanas significantly reduced FBG and serum MDA in patients with type 2 diabetes mellitus [34]. This study suggests that Hatha yoga exercise is as effective as conventional PT exercise in substantially improving biochemical, oxidative stress profile and antioxidant status in type 2 diabetic patients over a period of six months.

The beneficial effects of yoga on glycaemic control are well documented. In this study, Hatha yoga exercise significantly reduced FBG concentrations after six months. This result is in accordance with previous studies by Malhotra et al. who showed that yoga asanas significantly decreased FBG concentrations in type 2 diabetic patients after forty days [35]. A similar study by Jain et al. over the same time period demonstrated a significant reduction in hyperglycaemia measured by FBG and oral glucose tolerance [36]. Sahay et al. reported significant reduction in fasting and post-prandial blood glucose concentrations within three months of yoga exercise in type 2 diabetic patients [37]. Mercuri et al. found significantly decreased glycaemia after three months of yoga exercise in a similar group of patients [38]. The results of this study and others indicate the positive effect of yoga exercise on glycaemia control and suggest that such would be beneficial for the treatment of diabetes mellitus.

Lipoprotein abnormalities play an important role in the causation of diabetic atherosclerosis [39]. Dyslipidaemia causes morbidity and mortality in patients with type 2 diabetic mellitus and the most common pattern in type 2 diabetic patients is elevated triglyceride and LDL, and decreased HDL cholesterol concentrations [40]. The modifications of LDL lipoprotein increase atherogenicity and available data suggest that LDL is more atherogenic in individuals with type 2 diabetes mellitus [41]. In this study Hatha yoga and conventional PT exercises significantly reduced serum TC and VLDL concentrations with no significant change in triglyceride, LDL or HDL concentrations. Agrawal et al. reported significant improvement in glycaemic control and lipid profile in type 2 diabetic patients exposed to yoga exercise where there was significant reduction in serum TC, triglyceride and LDL concentrations associated with concomitant significant increase in HDL concentrations after three months [42]. Agte and Tarwadi observed statistically significant reduction by *Sudarshan kriya yoga *(SKY) on serum TC and triglycerides in type 2 diabetic patients after four months [43]. These authors suggested a promising potential for SKY as a complementary treatment for patients with diabetes [43]. The results of this study and others point to benefits for persons with diabetes mellitus with relationship to the risks associated with dysfunction of the lipid profile such as: macrovascular complications, endothelin-1 [44], coronary heart disease and oxidative stress [45].

Diabetic patients have been generally described as having high levels of oxidative stress [46]. Oxidative stress generally causes damage to the membrane polyunsaturated fatty acids leading to the generation of MDA, a thiobarbituric acid reacting substance (TBARS). Increased lipid peroxidation products in diabetic subjects with vascular complications, have been reported [47]. Some authors have shown that high concentration of glucose may be associated with the presence of oxidative stress as reflected by the increase of intracellular lipoperoxides [48]. Serum MDA levels are higher in patients with newly diagnosed type 2 diabetes mellitus [49] and its concentration is elevated in poorly controlled type 2 diabetic patients [50]. The serum MDA concentrations in controls, which remained unaltered over the six month period was significantly higher than that of the managed exercise groups. Other researchers have found significant reduction in plasma MDA *by Sudarshan kriya yoga *(SKY) in type 2 diabetic patients after four months [43]. In our study the decreased concentration of MDA by the third and sixth months in type 2 diabetic patients in Hatha yoga and conventional PT exercise groups indicated that there was a reduction in lipoperoxidation diabetes mellitus. The control of glycaemia and the decreased lipid profile parameters using yoga exercises are important influences on the decrease of this oxidative stress parameter and provide more support for the rational of a possible protective effect of yoga exercise against oxidative stress in diabetics.

Protein oxidation, in contrast to lipid peroxidation, does not have the features of chain reactions. The plasma proteins destructed by peroxidation have a quite long period. Therefore, the evaluation of POX in plasma is a respected marker of free radical intensity [51]. Reactive oxygen species modify amino acid side chains of proteins such as arginine, lysine, threonine and proline residues to form protein carbonyls [52]. Carbonyl group formation is considered an early and stable marker for POX, and elevated protein carbonyl levels are detected in type 2 diabetes mellitus and well correlated with the complications of diabetes [53]. The POX non-significantly increased in diabetic patients in the managed exercise groups, with minor increase in the control group. This indicates carbonyl group formation and thus evidence of free radical modification of proteins over the six months [54].

Exercise is a major therapeutic modality in the treatment of diabetes mellitus [55]. Exercise training has been known to be effective in type 2 diabetes mellitus by increasing insulin sensitivity [56], and regular exercise can strengthen antioxidant defenses and may reduce oxidative stress [57]. Exercises including yoga postures have been shown to play a role in preventing type 2 diabetes [58]. The yoga postures are slow rhythmic movements which emphasize the stimulation of the organs and glands by easy bending and extensions which do not over-stimulates muscles but concentrate on glandular stimulation [59]. A major benefit of non-exhaustive exercise such as yoga is to induce a mild oxidative stress that stimulates the expression of certain antioxidant enzymes. This is mediated by the activation of redox-sensitive signaling pathways [60]. For example, gene expression of SOD is enhanced after an acute bout of exercise preceded by an elevation of NF-kappaB and AP-1 binding. An increase in *de novo *protein synthesis of an antioxidant enzyme such as SOD or catalase usually requires repeated bouts of exercise [61]. This could explain the increase in the activity of SOD in the Hatha yoga and conventional PT exercise groups at the end of six months.

Cellular intracellular enzymes such as SOD and catalase along with non-enzymatic antioxidants (glutathione) act as primary line of defense to cope with the deleterious effects of reactive oxygen species [62]. Superoxide dismutase detoxifies superoxide radicals and converts them to hydrogen peroxide which is further converted to water by catalase and glutathione peroxidase. Reduced scavenging of free radicals by SOD, decreased glutathione and decreased activity of catalase are associated with diabetes and vascular pathology [46]. Reduced capacities of antioxidant enzymes lead to increased oxidative stress in diabetes [63]. Turk et al. reported an increase in SOD activity and decreased catalase activity and suggested that these alterations may be owing to the compensatory mechanisms of the antioxidant system in type 2 diabetics [64]. In our study, evaluation of antioxidant status demonstrated significant increase in SOD activity and non-significant increase in catalase activity with a concomitant significant reduction in MDA in the Hatha yoga and conventional PT groups after six months. The improved antioxidant status due to these exercise regimens may point to adaptive response to oxidative stress reflecting free radical production and increased enzyme biosynthesis [65]. Furthermore, in oxidative stress when excessive superoxide formation may be accompanied by increased nitric oxide levels, elevated SOD activity may play a protective role in preventing cells from peroxynitrite formation [66].

Exercise intensity, for diagnostic or exercise prescription purposes, has been expressed in terms of oxygen consumption (VO_2_), heart rate (HR), and/or ratings of perceived exertion [67]. Maximal oxygen consumption (VO_2max_) is generally accepted as the criterion measure of cardio-respiratory capacity [68]. Accordingly, recommended intensity levels for particular purposes may be accurately expressed in terms of VO_2max_. One of the limitations of the study is the lack of investigation of this parameter and the measurement of lactate. There were budgetary constraints and the authors recognized that the measurement of VO_2max _is generally restricted to sophisticated research settings due to the specialized equipment required. In this study, heart rate and Rating of Perception Exertion was used as valid indicators of exercise intensity. Changes in VO_2max _during different types of yoga breathing practices (pranayama) have previously been reported. Ray and colleagues reported that there is significant increase in VO_2max _in healthy individuals who practiced yogic exercise one hour every morning (6 days per week) for six months [69]. Another study showed decreased VO_2max _during Ujjayi pranayama practice in subjects who were practicing yoga for more than six months [70]. Carroll and colleagues found no relationship between heart rate and VO_2max _although there was mild increase in blood lactate in a group of yoga practitioners with yoga experience of 3 – 36 months [71].

## Conclusion

The findings of the study demonstrate the efficacy of Hatha yoga exercise on fasting blood glucose, lipid profile, oxidative stress markers and antioxidant status in patients with type 2 diabetes. The response observed using Hatha yoga exercise in type 2 diabetic patients was similar to that of conventional physical training exercise. These findings suggest that Hatha yoga exercise has therapeutic preventative and protective effects in type 2 diabetes by decreasing oxidative stress. This may have direct impact on the use of Hatha yoga exercise as a safe therapeutic modality in diabetes mellitus.

## Competing interests

The authors declare that they have no competing interests.

## Authors' contributions

LAG, EYM, EMZ were responsible for the study concept, coordinating research activities, the development of method, analyzing the data and writing the manuscript. DAMcG, RY, YTPF, RLA-L and RRI were involved in literature search, statistical analysis, data interpretation and writing the manuscript. All authors read and approved the final manuscript.

## Pre-publication history

The pre-publication history for this paper can be accessed here:


